# Evaluating the Effect of Government Emission Reduction Policy: Evidence from Demonstration Cities in China

**DOI:** 10.3390/ijerph18094649

**Published:** 2021-04-27

**Authors:** Yunchan Zhu, Shuo Han, Yimeng Zhang, Qi Huang

**Affiliations:** 1 Economics and Management School, Wuhan University, Wuhan 430072, China; Zhangyimeng@whu.edu.cn; 2 School of Mathematics and Statistics, Wuhan University, Wuhan 430072, China; 2018302060281@whu.edu.cn; 3 KoGuan School of Law, Shanghai Jiao Tong University, Shanghai 200030, China; huangqi23@sjtu.edu.cn

**Keywords:** emission reduction policy, industrial SO_2_ emission, industrial wastewater emission, green development

## Abstract

The effectiveness of government environmental policies is pivotal to environmental quality and provides the reference for further policy design. This paper estimates the effect of comprehensive demonstration of fiscal policy for ECER (Energy Conservation and Emission Reduction) on pollution emissions in Chinese cities with the sample period from 2003 to 2016, which is an important practice for policy integration. We find that this policy reduces the industrial SO_2_ (sulfur dioxide) emission by 23.8% on average and the industrial wastewater emission by 17.5% on average. This policy, implemented by Chinese government, has effectively achieved its target for emission reduction. A series of robustness checks are also conducted to verify the baseline results. Mechanism analysis indicates that this policy has the effect by the change in the industry structure and the enhancement of fiscal capacity, especially the capacity of fiscal revenue. Some policy recommendations, such as laying emphasis on the policy integration, integrating the financial resources of governments and expanding the demonstration effect, are proposed in order to facilitate green development in Chinese cities.

## 1. Introduction

Environmental issues have become a major concern around the world. Pollution generates several negative externalities, which result in the inconsistencies between social cost and private cost, social benefit and private benefit [1,2,3]. Therefore, the environmental problems cannot be effectively solved only by the market mechanism, and the government should adopt additional policies to control pollution. The choice of government policy tools for emission reduction and the real effects of target-based environmental policy are important topics to consider in order to promote the urban green development and the sustainable development of all the countries and regions.

In the current work, we estimate the effect of comprehensive demonstration of fiscal policy for ECER (Energy Conservation and Emission Reduction) on pollution emissions in Chinese cities. There are three reasons for this research. First, this policy is an active exploration of government environmental regulation, from single policy to policy integration, and has important practical value. Examination of the effectiveness of target-based and comprehensive policy provides a reference for further environmental governance. Second, Chinese government has given priority to pollution prevention and control, which has been one of the three critical challenges facing in all the society in recent years [4]. Evaluation of the effect of the policy aimed at emission reduction in the largest developing country is obviously a significant and remarkable endeavor. Third, according to the environmental performance index reported by Yale University, Columbia University and the World Economic Forum, China usually occupies a very low position in the world rankings, and was even ranked the last fourth in terms of air quality in 2018 (these data could be collected from the website https://epi.yale.edu/ (accessed on 25 October 2020). There is a sharp contrast between the Chinese GDP ranking and its environmental performance index ranking. It is important to analyze whether the environmental policy meets the demands of the current economic structure and contributes to urban sustainable development.

Our study contributes to two strands of literature: studies regarding government environmental policies as well as the connected decision problems, and studies that examine the effects of public policies and regulations, especially fiscal policy, to address the negative externalities that are generated by pollution emissions.

Generally, three types of environmental policies are used to save energy, reduce emissions and promote green growth: command-and-control, the market-based and the informal (also called voluntary) policies [5,6]. The first two have been widely adopted in many countries and include emission standards, pollution regulations, environmental taxes, pollution fees and so on. The last type is not imposed by the government but instead depends on public awareness. For instance, the press can act as an informal regulator [7]. In this study, we mainly focus on the literature regarding environmental policies imposed by the government in this study. Government, as the public sector, has the duty to implement policy strategies, such as the adoption of clean energy technologies, to balance economic development with environmental protection [8,9]. The effects of environmental policies appear to differ between countries or regions [10,11].

Much attention has been paid to the impact of environmental regulation and standards, such as pollutant control policy, environmental courts and fuel standards. Chen et al. (2018) [12], and Chen et al. (2017) [13], analyzed the impact of the Two Control Zone (the control of acid rain and the emission of sulfur dioxide in targeted areas) policy in China. The findings indicated a significant decrease in SO_2_ emissions, and the stricter environmental regulation resulted in a reduction in polluting activities. Barreca et al. (2017) [14], examined the effect of an acid rain program in the United States, and found that a permanent decrease in pollution and relative mortality in treatment counties. However, Wang et al. [15], found that environmental policy stringency had a weak impact on both PM_2.5_ emission based on the panel data for 23 OECD countries. The environmental court and jurisprudence have evolved over during the past three decades, which has increased the integrity of environmental justice, improved the trial efficiency of environmental cases and imposed strict constraints aimed at saving resources and reducing pollution [16,17]. Zhang et al. (2019) [18], evaluated the effect of the establishment of Chinese environmental courts and found that the policy increased air quality significantly at the city level. As for gasoline standards, the results were contradictory in various countries. Auffhammer and Kellogg (2011) [19] found that US federal gasoline standards did not improve air quality. However, the opposite was observed in China. Li et al. (2020) [20], showed that the enforcement of Chinese gasoline standards improved air quality significantly, especially in terms of fine particles and ozone. Some literature [20,21,22,23,24,25] also focused on the road transport policies and driving restrictions, and found that the effects of these policies are different across various cities or countries.

However, there are few similar studies focusing on the effect of comprehensive demonstration of fiscal policy for ECER conducted in China, which is a new practice for policy integration. This paper uses Chinese prefecture-level data from 2003 to 2016 and considers the list of demonstration cities for the empirical estimation. The results show that this government emission reduction policy significantly reduces the industrial SO_2_ and the industrial wastewater emissions by 23.8% and 17.5% on average, respectively; thereby improving environmental quality and achieving the initial policy goals. Therefore, this study enriches previous related literature about the effect of public environmental policies. We construct a DD (difference-in-difference) estimation framework, which could address the endogenous issues and improve the accuracy of the estimation. Some robustness checks are conducted to verify the results as well. The quantitative analysis of the effectiveness of the government policy provides evidence for the choice of further policies. This research also provides a reference by which other countries and regions can understand the role of emission reduction policies in urban environmental protection. These findings will be useful for the policy makers seeking to devise more effective policies.

The remainder of this paper is organized as follows. Section 2 introduces China’s comprehensive demonstration of fiscal policy for ECER. Section 3 provides the framework for empirical estimation and introduces the data sets. Section 4 reports the results and discusses the underlying mechanism. The last section offers some conclusions.

## 2. China’s Comprehensive Demonstration of Fiscal Policy for ECER

Chinese government proposed the comprehensive demonstration of fiscal policy for ECER (Energy Conservation and Emission Reduction) for the first time in June 2011. The Ministry of Finance and the National Development and Reform Commission of China issued the “Notice on Carrying out Comprehensive Demonstration of Fiscal Policy for Energy Conservation and Emission Reduction” and decided to implement the comprehensive demonstration of fiscal policies for energy conservation and emission reduction in particular cities during the twelfth Chinese five-year plan period (2011–2015). Eight cities were finally selected as the first group of demonstration cities [26]. The local government is mainly responsible for this policy, and the demonstration city acts as a platform to increase the integration of various fiscal policies regarding energy conservation and emission reduction. This policy is from the “point” to the “face” (from local to overall or from small to large scale), from single policy to policy integration, so it places full emphasis on the role of fiscal policy for energy conservation and emission reduction. It is expected that the emission reduction targets could be realized by accelerating the innovation of the system and mechanism, actively optimizing the economic structure and promoting the economic transformation.

The second series of comprehensive demonstrations was announced in 2013, and 10 cities were selected as the second group of demonstration cities [27]. The third group of demonstration cities included 12 cities; this was the final group, and the demonstration cities were not be expanded further [28]. The details of the three groups of demonstrations cities of three batches are listed in Table 1.

The comprehensive demonstration of fiscal policy for ECER mainly includes six aspects: the first focuses on reducing carbonization in industry, to speed up the adjustment of the industrial structure and the development of strategies for the emerging industry. It is aimed at resolutely eliminating the outdated production capacity, supporting key enterprises in implementing energy-saving technological transformation and promoting the application of advanced the green technologies. The second aspect involves renovating the urban transportation system around clean transportation, increasing the use of new energy vehicles, encouraging residents to prioritize public transportation and advocating for green travel throughout society. The third involves promoting the development of energy-efficiency and green buildings. The fourth is to accelerate the development of the service industry, centered on intensification, with a focus on creating service industry circles (belts) or parks. The fifth aspect focuses on the reduction of major pollutants to improve urban environmental quality, build a supporting pipeline network for urban sewage-treatment facilities and develop a robust circular economy. The sixth is to optimize the urban energy structure, focusing on the large-scale utilization of renewable energy.

The implementation of comprehensive demonstration of fiscal policy for ECER aims to transform the existing single environmental policies into an integrated policy. The completion of targets for energy conservation and emission reduction is an essential requirement in order to ensure a comprehensive demonstration and is also an important component of the performance evaluation for the local governments. In this paper, we investigate the emission reduction effect of this policy and provide a reference for the effectiveness of the target-based performance evaluation.

## 3. Estimation Strategy

### 3.1. Estimation Framework

The main issue discussed in this paper is whether the comprehensive demonstration of fiscal policy for energy conservation and emission reduction has effectively reduced urban environmental pollution. In order to solve the endogenous problems commonly faced in the previous literature, we construct a difference-in-difference model by regarding this policy as a quasi-natural experiment: the first level of difference is from the city, and the second level of difference is from the year. Specifically, the demonstration cities of this policy were announced in three groups and thirty cities were selected in total during the years of 2011, 2013 and 2014. The cancellation of the Haidong region and the establishment of the prefecture-level Haidong city took place in 2013, and a large amount of data relating to the period before 2013 were missing, so Haidong city was not included in the sample for this research. Therefore, we choose the remaining 29 cities as the treatment group. As for the control group, following Tan et al. (2018) [29], we choose the cities that were geographically adjacent to the treatment group but not included in the treatment group. The DD method in this paper compares the differences in pollutant emissions between demonstration cities and non-demonstration cities before and after this policy is implemented. The baseline DD estimation was the following specification:

(1) $$y_{it}=\alpha_{0}+\beta policy_{it}+\gamma Z_{it}+\eta_{i}+\delta_{t}+\varepsilon_{it}$$

where *i* and *t* indicate city and year, respectively; the dependent variable *y_it_* represents the pollution emissions, which includes the logarithm of total volume of industrial SO_2_ (the unit of which is ton) and total volume of the industrial wastewater (the unit of which is 10,000 tons). *Z_it_* indicates a vector of control variables, such as the level of economic development, industrial structure, technological innovation, openness, population scale and urban greening rate; *η_i_* is the city-fixed effect, controlling for the unobserved, time-unvarying city attributes that might affect the pollution emissions; *δ_t_* is the year-fixed effect, controlling for nation-wide shocks in a particular year likely to influence all cities in a similar manner; and *ε_it_* is the error term.

Here, *policy_it_* is the regressor we are interested in, which is a dummy variable indicating the policy status of city *i* in year *t*. Specifically, *policy_it_ = treated_i_* × *post_it_*, where *treated_i_* is set to 1 if the city *i* was selected as a comprehensive demonstration cities of fiscal policy for ECER during the sample period, and set to 0 otherwise. Here, *post_it_* is a post-treatment variable, taking the value of 1 if the city *i* has adopted this policy and 0 otherwise. We cluster the standard errors at the city level as well, to address the potential problems of heteroskedasticity and serial correlation.

Here, *β* and *γ* are coefficient vectors to be estimated. The key coefficient is *β*, which is also referred to as the DD estimator, capturing the average effect of this policy on pollution emissions. The DD method could accurately evaluate the impact of the policy by eliminating the influence of differences in various cities between the treatment group and the control group, and then forming a causal inference of the implementation of the policy. If *β* is significantly negative, we deem that this policy, implemented by Chinese government, exerted the expected emission reduction effect as expected.

### 3.2. Data

To analyze whether the comprehensive demonstration of fiscal policy for ECER conducted by Chinese government has effectively reduced urban pollution emissions, we mainly use three main data sets, which include the indicators of the implementation of this policy in the demonstration cities, the pollution emissions and other influencing factors for the sample cities. The sample period in the empirical analysis is from 2003 to 2016.

We manually collected the list of demonstration cities which were selected for the comprehensive demonstration, and we also collected the time of the policy implementation. The data were collected primarily from websites of the Ministry of Finance and the National Development and Reform Commission of China, as mentioned in Section 2.

Air pollution and water pollution are the two main types of urban pollution. Considering the targets of this policy, the availability of annual data in prefecture-level cities and following the related literature on the urban pollution emissions [30], the measurement of pollution emissions in this paper contains the industrial sulfur dioxide emission and the industrial wastewater emission. We carried out logarithmic processing on these variables, to ensure the stability of the data and facilitate the estimation and comparison presented in Section 4. The data were collected from the *China City Statistical Yearbook* published by the Urban Social and Economic Investigation Department of the National Bureau of Statistics in China.

In order to control the possible influence of other variables on urban pollution emissions, some control variables are defined as follows:

(1) The level of urban economic development. The economic development may have the multiple influences on pollution emissions [31,32,33]. On the one hand, the higher the level of urban economic development, the more production, which will bring about more pollution emissions, so economic growth might serve to pollution increase. On the other hand, as a result of the economic growth, local government has more financial resources to invest in environmental protection and pollution control, which contributes to the control of pollutant emissions and the improvement of environmental quality. We use the logarithm of real gross regional domestic product to present the level of economic development.
(2) Technological innovation. Technological innovation plays an important role in environmental protection and the application of green technology for environmental protection could reduce emissions [34]. We use the number of patent applications which contain inventions, utility models and designs in the city to measure technological innovation, and we obtained the data from the Chinese Research Data Services (CNRDS) platform.
(3) Openness. For developing countries, opening up will help to introduce foreign advanced technology, enhance environmental protection awareness and improve environmental quality; however, some scholars have suggested that trade will cause environmental degradation due to the “pollution haven” hypothesis [35,36]. The total import and export of China has grown from 1.13 billion dollars in 1950 to 4.6 trillion dollars in 2018, rendering it the largest trading country. China absorbed 138.3 billion dollars in foreign capital, ranking second in the world [37]. Thus, we choose the annual amount of foreign capital actually used by the city in order to analyze the impact of openness on the pollution emissions.
(4) Population scale. Considering the heterogeneity of population scale in different cities, so we control the influence of the population factor using the logarithm of average annual population. The relationship between the population factor and pollution emission is uncertain. A larger population scale usually means a higher degree of industrialization and urbanization, and in turn, more environmental pollution is discharged in that city [38]. Conversely, population concentration may also help to achieve more efficient energy use due to increasing returns to scale. In addition, residents of large cities or economically developed areas are often more aware of environmental pollution, meaning that they are more willing to improve environmental quality [39].
(5) Industrial structure. The secondary industry, including high-energy and high-pollution industries, emits a large amount of pollution. Compared with the secondary industry, the tertiary industry generally generates lower pollution emissions. The industrial structure is obviously an important factor affecting the urban pollution emissions [40,41]. We use the proportion of the secondary industry to indicate the industrial structure [42]. It is expected that the higher the proportion of the secondary industry, the more serious the pollution emissions will be.
(6) Greening rate. Some studies have proven that the urban greening is conducive to greater environmental quality [43]. We use the greening coverage rate of built-up areas, which is the percentage of green areas to urban built-up areas, to represent the greening rate.

**Figure 1:**
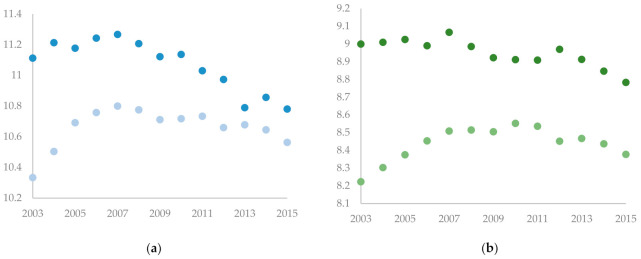
The evolution of the averaged variables. (**a**) shows the year-average ln (*SO_2_*) vs. year. The scatter plot of dark color represents the treatment group, and the scatter plot of light color represents the control group. (**b**) shows the year-average ln (*wastewater*) vs. year. The scatter plot of dark color represents the treatment group, and the scatter plot of light color represents the control group.

**Figure 2:**
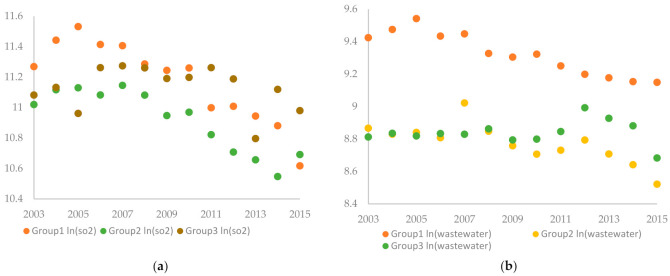
The evolutions of the year-average ln (*SO_2_*) and the year-average ln (*wastewater*) by group. Group 1, Group 2, Group 3, respectively, represents the first, the second, the third group of demonstration cities. (**a**) shows the year-average ln (*SO_2_*) vs. year, and (**b**) shows the year-average ln (*wastewater*) vs. year.

The data on the control variables were mainly collected from the *China City Statistical Yearbooks* during the sample years, supplemented by the province-level statistical yearbooks or the database of the CNRDS platform. Table 2 introduces the definition of variables, and Table 3 presents the descriptive statistics of the key variables. The evolutions of the year-average ln (*SO_2_*) and the year-average ln (*wastewater*) are shown in Figure 1 and Figure 2.

## 4. Results and Discussion

We use the DD approach to estimate the effect of the comprehensive demonstration of fiscal policy for ECER on urban pollution emissions. First, the baseline estimates are conducted as the Equation (1). Next, we do a series of robustness checks, including the test on parallel trend assumption, substitution of the variable, the adjustment of the sample period and sample size, and the placebo test. Lastly, we discuss the results and analyze the channels underlying the estimated effect of this government emission reduction policy.

### 4.1. Baseline Estimates

We first employ the DD method to test the effect of this policy on pollution emissions. The baseline estimation results are reported in Table 4. The dependent variable of the first and second column is ln (*SO_2_*) (the logarithm of the industrial SO_2_ emissions), and the dependent variable of the third and fourth column is ln (*wastewater*) (the logarithm of industrial wastewater emissions). Column (1) and column (3) present the regression without the control variables, and the control variables are added in the regression of column (2) and column (4). The results show that the coefficient of *policy* is negative and statistically significant at the 1% level in all the columns. The coefficient of *policy* is −0.238 in the column (2) after controlling other influencing factors, which indicates that the comprehensive demonstration of fiscal policy for ECER reduces the industrial SO_2_ emissions by 23.8% on average. The coefficient of policy is −0.175 in the column (4) after adding the control variables, which indicates that this policy reduces the industrial wastewater emissions by 17.5% on average. To summarize, these results indicate that this government emission reduction policy has realized the expected outcome, demonstrating a significantly negative effect on the pollution emissions. We also estimate the effect of this policy on some other pollution emissions such as industrial chemical oxygen demand, industrial ammonia nitrogen and PM_2.5_ (fine particles with a diameter of 2.5 μm or less) in Table A1 in Appendix A, which also indicates the positive effects of applying the policy manifested in a significant reduction in the industrial ammonia nitrogen and industrial chemical oxygen demand.

### 4.2. Robustness Checks

#### 4.2.1. Test on the Parallel Trend Assumption

We cannot use the traditional method to test the assumption of the parallel trend (also referred to as the common trend) of DD estimation, due to the inconsistent time for the implementation of this policy in the demonstration cities. Based on this, following the previous literature on testing strategy for the parallel trend of a multi-period DD model [44,45,46], we construct the following method of event study to test the assumption:

(2) $$y_{it}=\alpha_{0}+\beta_{k}\sum_{k\leq -4}^{4+}D_{itk}+\gamma Z_{it}+\eta_{i}+\delta_{t}+\varepsilon_{it}$$

In the Equation (2), we mainly use $\sum_{k\leq -4}^{4+}D_{itk}$ instead of $policy_{it}$ in the Equation (1), and the specification of other variables is the same as in the Equation (1). $\sum_{k\leq -4}^{4+}D_{itk}$ indicates whether the sample is in the *k* year after the policy implementation (if k is negative, it means that the sample is in the *k* years before the policy implementation). If the sample is in the *k* year, $D_{itk}$ is set to 1, and 0 otherwise. If the results find that the difference in pollution emissions between the treatment group and the control group is mainly caused by the post-policy $D_{itk}$ (k > 0) rather than the factors before the reform (k < 0), then it could be quantitatively determined that the treatment group and the control group conform to the assumption of the parallel trend and the regression result of Equation (1) has no obvious selection bias.

**Figure 3:**
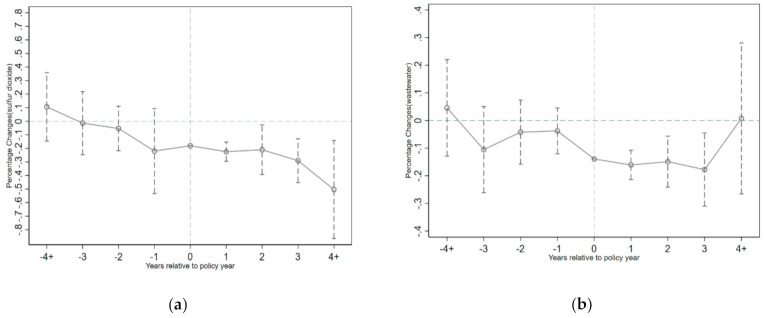
The dynamic effect of policy on the pollution emissions. Note: (**a**,**b**) plot the impact of this policy on the logarithm of the industrial SO_2_ and wastewater emissions, respectively. The dashed lines represent 95% confidence intervals, adjusted for city-level clustering.

It can be seen from Figure 3 that whether the dependent variable is the industrial SO_2_ or the industrial wastewater emissions, the regression coefficient of the previous periods before the implementation of this policy fluctuates around 0 and there is no significant trend of change at least at the 5% statistical level. The results indicate that the dependent variables of the treatment group and the control group display common time trends before the year of the policy’s implementation. In addition, for SO_2_ in Figure 3a, most of the coefficients are significantly different from 0 after the implementation of the policy, which also reflects the fact that the effect of this government emission reduction policy is dynamic and continuous. For wastewater in Figure 3b, the effect of policy disappears in the fourth year after the policy’s implementation. In conclusion, the results in Figure 3 prove that the parallel trend assumption is satisfied, so the results in Table 4 are reliable.

#### 4.2.2. Variable Substitution

The method of variable substitution is one of the methods of robustness checks that is widely used in the previous literature. We successively change the indicators of *policy* variable and control variable to test the robustness of the results in Table 5.

The analysis of the baseline estimate is based on the implementation year of the policy, so that different policy implementation times for different groups of cities are not distinguished. For this, we further refine the *policy* variables to the monthly level. If the policy is implemented in the demonstration city *i* in the *n*th month of the year *t*, and the corresponding variable is $policy_{it}^{*}=\left(12-n+1\right)/12$, and it equals 1 in the year *t + 1*. Specifically, if the policy implementation time is in January, the variable $policy_{it}^{*}=\left(12-1+1\right)/12=1$; if the policy implementation time is in December, the variable $policy_{it}=\left(12-12+1\right)/12=1/12$. The regression results after replacing the policy variable are shown in column (1) of Table 5. After changing the measurement of the status variable of the policy implementation from year to month, we find that the effect of comprehensive demonstration of fiscal policy for ECER on the industrial SO_2_ and the industrial wastewater is still significantly negative. In addition, the degree of influence is greater, which proves the reliability and the validity of the baseline results.

Invention patent is the best indicator of substantive innovation; thus, we replace ln (*patent*) with ln (*invention*) in the column (3) and column (4) of Table 4. The coefficients of *policy*, −0.233 and −0.179, are close to those in the column (2) and column (4) of Table 4. Therefore, these robustness checks absolutely support the results in Table 4.

#### 4.2.3. Sample Adjustment

Adjustment of the sample is another commonly-used method to check robustness which mainly involves changing the sample period and the sample size. We adjust the sample period to 2007–2016 to shorten the time window, and we eliminate the data for the period before 2007 in the column (1) and column (2) in the Table 6. Shortening the time window serves to eliminate the impact of other policies as much as possible. The results show that the coefficients of *policy* are still negative and statistically significant at the 1% level, which verifies the emission reduction effect of this policy. In the column (3) and column (4), we change the sample size by removing the cities that are adjacent to more than one city in the treatment group, to test whether the baseline conclusions are still robust. The coefficients of *policy* in the new regressions are similar to those in Table 4, indicating that the baseline results are robust.

#### 4.2.4. Placebo Test

Research on DD model usually uses two methods, including changing the treatment group or the policy implementation time, to conduct placebo test, which is also called the counterfactual test. In this part, we conduct a placebo test by randomly assigning the comprehensive demonstration cities of fiscal policy for ECER, in order to test the extent to which the results are affected by the omitted variables [46]. More specifically, the virtual demonstration cities are randomly selected from all the sample cities, and the virtual policy implementation time of different cities is also randomly assigned during the sample period, so the *policy* variable is randomly generated. Given the random data generation process (DGP), the false *policy* variable should have produced no significant estimate with a magnitude close to zero, that is, the expected value of the estimated coefficient is around 0 due to random assignment for the treatment and control groups; otherwise, it would indicate a misspecification of the DD estimation. In order to increase the identification power, this placebo test is repeated 500 times.

**Figure 4:**
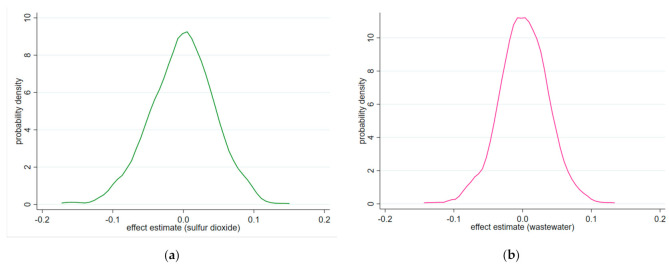
Distribution of estimated coefficients of placebo test. Note: (**a**,**b**), respectively, show the cumulative distribution density of the estimated coefficients of ln (*SO_2_*) and ln (*wastewater*) is from 500 simulations randomly assigning the *policy* status to cities.

The results of these falsification tests are shown in Figure 4. Both Figure 4a,b show that the distribution of the emission reduction effect estimates from random assignments is clearly centered around zero, which indicates that there is no effect on the randomly constructed demonstration cities of ECER policy. Meanwhile, Figure 4a reports that the benchmark coefficient of ln (*SO_2_*), −0.238, from column (2) in Table 4 is located outside the entire distribution of the estimated coefficients of the falsification test. Figure 2b reports that the benchmark coefficient of ln (*wastewater*), −0.175, from column (4) in Table 4, is far from the distribution of the estimated coefficients from the 500 runs. Collectively, these results suggest that the negative and significant effect of the comprehensive demonstration cities of fiscal policy for ECER on pollution emissions is not driven by the unobserved factors. Therefore, the placebo test supports the estimation strategy and indicates that the baseline estimates in Table 4 are robust.

### 4.3. Mechanism Interpretation

In this section, we discuss the mechanism (or channels) underlying the negative links between the comprehensive demonstration of fiscal policy for ECER and pollution emissions. We assumed that this policy could reduce emissions by these two channels: (1) upgrading of urban industry structure, and (2) incresing government regulation, especially the fiscal capacity of the local government.

#### 4.3.1. Industry Structure

According to the implementation goals of the comprehensive demonstration of fiscal policy for ECER, this policy aims to guide industrial innovation and the upgrading of industrial structure, thereby reducing pollution emissions. As mentioned above, compared with the secondary industry, the tertiary industry generally generates lower pollution emissions. The change in industrial structure will influence the urban pollution emissions [40,41]. The traditional industries could achieve the green upgrades through technological transformation, and other industries could develop green industries through the optimization and adjustment of industrial structure, to promote the industrial upgrading and the transformation of the development mode of the overall city.

In order to test this mechanism, this paper measures the change in urban industrial structure via the ratio of the secondary industry (*si*) and the ratio of the tertiary industry (*ti*). The results are reported in Table 7. It can be seen that the coefficients of *policy* in column (1) and (2), of which the dependent variable is the ratio of the secondary industry, indicating that the comprehensive demonstration of fiscal policy for ECER has significantly reduced the proportion of secondary industry. The regressions of the impact on the ratio of tertiary industry are listed in column (3) and (4), which show that the proportion of secondary industry in the demonstration cities after the implementation of this policy. These results proved the first channel of change in industry structure.

#### 4.3.2. Fiscal Capacity

This government emission reduction policy is essentially a fiscal policy, and fiscal tools are mainly used to achieve the targets. According to the statistics, the central government had allocated 4 billion yuan as the comprehensive award funds to the eight demonstration cities of the first group by the end of 2012, and the corresponding local governments at province-level and prefecture-level had also allocated more than 20 billion yuan in total. These eight demonstration cities continued to receive a comprehensive award fund of 4 billion yuan in 2013 [26]. At the same time, the award funds were allocated by the factor method. Factors include workload, the effect of energy saving and emission reduction and long-term mechanism construction, each with a certain weight. Therefore, the comprehensive demonstration of fiscal policy for ECER increases the fiscal revenue of the demonstration cities, and increases the expenditure pressure for energy saving and emission reduction as well. If the fiscal capacity increases, the policy’s target for emission reduction of policy is achieved.

In Table 8, we examine the second mechanism of fiscal capacity. The results in column (1) and column (2) show that this policy has a positive impact on both the fiscal expenditure and fiscal revenue, and the coefficient of the effect on the fiscal revenue is slightly higher. This means that the local government has more financial resources for energy conservation and emission reduction due to the comprehensive demonstration policy. Furthermore, we consider the fiscal gap, which is usually used to measure the fiscal pressure of the government [47]. The method of measuring the fiscal gap is as follows: *gaprate* = (fiscal expenditure-fiscal revenue)/fiscal revenue. The result of regression on the fiscal gap is presented in the column (3) of Table 8. The coefficient of *policy* is negative, indicating that this policy might reduce the fiscal gap, but it is not statistically significant. In conclusion, the second channel is that this policy could achieve the effect of emission reduction through the enhancement of fiscal capacity, especially the capacity of fiscal revenue.

## 5. Conclusions and Policy Implications

Pollution control has been one of the three critical challenges of Chinese government in recent years, and pollution issues have become a major concern throughout society. This paper focuses on the effect of the government emission reduction policy, and estimates the impact of comprehensive demonstration of fiscal policy for ECER on the pollution emissions. Base the data of Chinese cities from 2003 to 2016, we find that this policy reduces the industrial SO_2_ emission by 23.8% on average and the industrial wastewater emission by 17.5% on average; that is, the emission reduction policy implemented by Chinese government has achieved its target for pollution control. Some robustness checks are conducted to verify the baseline results. The test on the parallel trend assumption, variable substitutions, changing the sample period and placebo test all support the conclusion that this policy significantly reduce pollution emissions. As for the mechanism analysis, we find that the underlying channels are industry structure and fiscal capacity. Specifically, the implementation of this policy could reduce the pollution emissions by decreasing the proportion of the secondary industry and increasing the proportion of the tertiary industry. The target for emission reduction could be achieved by another channel, namely the enhancement of fiscal capacity, especially the capacity of fiscal revenue. These findings could be explained by the effectiveness of the targeted-based comprehensive policy and performance evaluation system. Our study also provides a useful reference for promoting green development and sustainable development in Chinese cities. Based on these valuable results presented above, we suggest the following policy implications:

(1) Emphasis should be placed on the policy integration, and the systematic and comprehensive promotion of pollution prevention and control. The results show that the comprehensive demonstration reduced pollution emissions significantly. Government should comprehensively consider fiscal policy, urban planning and spatial layout and further integrate the pollution control, energy structure adjustment and green development mode in a deeper degree.
(2) The financial resources of governments should be integrated, effectively avoiding the overlap or blind spots in policies. Based on the platform of the city, the fiscal funds of the central and local government should be integrated and used, effectively avoiding overlapping of funds. At the same time, the central government should provide comprehensive funds for emission reduction, and the cities should use them in a coordinated manner, which enables the existing policies to be fully connected and benefit from the synergy of funds.
(3) The demonstration effect should be expanded, and the effective practices and measures should be promoted in the non-demonstration cities. The demonstration cities should continue to implement and improve this policy, and summarize its success during the process of policy implementation. The non-demonstration cities should attach importance to the effectiveness of this policy and actively explore additional new policy tools by taking the demonstration cities as a benchmark.

## Figures and Tables

**Table 1:** Demonstration cities of ECER (Energy Conservation and Emission Reduction) fiscal policy.

Year	Cities
2011	Beijing, Shenzhen, Chongqing, Hangzhou, Changsha, Guiyang, Jilin, Xinyu
2013	Shijiazhuang, Tangshan, Tieling, Qiqihaer, Tongling, Nanping, Jingmen, Shaoguan, Dongguan, Tongchuan
2014	Tianjin, Linfen, Baotou, Xuzhou, Liaocheng, Hebi, Meizhou, Nanning, Deyang, Lanzhou, Haidong, Wulumuqi

Note: The list of model cities was retrieved from the Chinese government’s websites.

**Table 2:** Definition of variables.

Variable	Defination of Variables	Unit
In (*SO_2_*)	industrial sulfur dioxide emission (logarithm)	ton
ln (*wastewater*)	industrial wastewater emission (logarithm)	ten thousand ton
ln (*grp*)	real gross regional domestic product (logarithm)	ten thousand yuan
ln (*patent*)	the number of patent applications (logarithm after adding one)	-
ln (*open*)	the annual amount of foreign capital actually used (logarithm)	ten thousand dollar
ln (*pop*)	average annual population (logarithm)	ten thousand
*indus*	the proportion of the secondary industry	%
*greenrate*	the percentage of green areas to urban built-up area	%

**Table 3:** Summary statistics of the key variables.

Variable	Full Sample				Treament Group				Control Group			
	Mean	Sd	Min	Max	Mean	Sd	Min	Max	Mean	Sd	Min	Max
ln (*SO_2_*)	10.69	1.003	4.159	13.43	11.01	0.975	7.147	13.43	10.61	0.993	4.159	13.12
ln (*wastewater*)	8.521	1.043	5.081	11.42	8.925	1.026	5.759	11.42	8.413	1.022	5.081	11.37
ln (*grp*)	16.03	1.046	13.10	19.36	16.47	1.225	13.10	19.36	15.91	0.959	13.24	19.09
ln (*patent*)	6.491	1.801	1.792	12.02	7.303	1.908	2.398	12.02	6.275	1.708	1.792	11.71
ln (*open*)	9.662	1.842	2.773	14.94	10.46	1.828	5.438	14.94	9.447	1.787	2.773	13.93
ln (*pop*)	5.964	0.608	4.261	8.129	5.998	0.812	4.261	8.129	5.955	0.541	4.368	7.244
*si*	0.487	0.100	0.027	0.859	0.486	0.101	0.193	0.747	0.487	0.0998	0.0266	0.859
*greenrate*	0.376	0.184	0.004	3.866	0.431	0.352	0.0555	3.866	0.361	0.0929	0.00380	0.952
**Variable**	**Group 1**				**Group 2**				**Group 3**			
	**Mean**	**Sd**	**Min**	**Max**	**Mean**	**Sd**	**Min**	**Max**	**Mean**	**Sd**	**Min**	**Max**
ln (*SO_2_*)	11.10	1.084	8.327	13.43	10.86	1.008	8.380	12.71	11.08	0.841	7.147	12.39
ln (*wastewater*)	9.309	1.053	7.594	11.36	8.737	1.263	5.759	11.42	8.813	0.604	7.552	10.31
ln (*grp*)	17.26	1.262	13.90	19.36	15.98	1.142	13.10	18.04	16.35	0.975	14.02	19.00
ln (*patent*)	8.653	2.008	3.932	12.02	6.553	1.654	2.398	10.82	7.003	1.512	4.043	11.17
ln (*open*)	11.79	1.567	8.220	14.08	9.881	1.618	5.438	13.18	9.996	1.688	5.714	14.94
ln (*pop*)	6.310	0.958	4.695	8.129	5.682	0.799	4.261	6.945	6.059	0.577	4.986	6.951
*indus*	0.460	0.100	0.193	0.670	0.491	0.104	0.270	0.747	0.499	0.0977	0.286	0.717
*greenrate*	0.413	0.0737	0.181	0.689	0.517	0.578	0.0841	3.866	0.365	0.0712	0.0555	0.583

Note: Mean, Sd, Min and Max denote the mean value, standard deviation, minimum value and maximum value of the variable, respectively. Group 1, Group 2, Group 3, respectively, represents the first, the second, the third group of demonstration cities. The italics are for the indicators of variables in all tables.

**Table 4:** Effect of the policy on pollution emissions (baseline estimates).

Variable	(1)	(2)	(3)	(4)
	ln (*SO_2_*)	ln (*SO_2_*)	ln (*Wastewater*)	ln (*Wastewater*)
*policy*	−0.278 ***	−0.238 ***	−0.146 ***	−0.175 ***
	(0.0542)	(0.0551)	(0.0366)	(0.0374)
ln (*grp*)		−0.0747		0.300 ***
		(0.130)		(0.1000)
ln (*patent*)		−0.0611 **		−0.0689 **
		(0.0292)		(0.0276)
ln (*open*)		−0.0304 *		−0.0135
		(0.0175)		(0.0152)
ln (*pop*)		−0.110		0.0803
		(0.207)		(0.172)
*indus*		1.020 ***		0.429
		(0.347)		(0.274)
*greenrate*		−0.120 ***		−0.0468
		(0.0449)		(0.0428)
No. of observations	1906	1848	1907	1850
R-squared	0.811	0.815	0.877	0.875
City fixed effect	YES	YES	YES	YES
Year fixed effect	YES	YES	YES	YES

Note: The robust standard errors are reported in parentheses. All regressions control for the city fixed effects and the year fixed effects. Superscripts ***, **, * denotes significances at the 1%, 5%, 10% levels, respectively.

**Table 5:** Robustness checks—variable substitution.

Variable	(1)	(2)	(3)	(4)
	ln (*SO_2_*)	ln (*Wastewater*)	ln (*SO_2_*)	ln (*Wastewater*)
policy *	−0.646 ***	−0.460 ***		
	(0.133)	(0.0938)		
*policy*			−0.233 ***	−0.179 ***
			(0.0548)	(0.0374)
ln (*grp*)	−0.0506	0.317 ***	−0.101	0.294 ***
	(0.130)	(0.101)	(0.130)	(0.102)
ln (*patent*)	−0.0634 **	−0.0702 **		
	(0.0291)	(0.0276)		
ln (*open*)	−0.0298 *	−0.0131	−0.0296 *	−0.0131
	(0.0175)	(0.0152)	(0.0174)	(0.0152)
ln (*pop*)	−0.0826	0.0992	−0.118	0.0943
	(0.197)	(0.175)	(0.204)	(0.183)
*indus*	0.969 ***	0.462 *	1.054 ***	0.421
	(0.346)	(0.275)	(0.356)	(0.285)
*greenrate*	−0.116 ***	−0.0437	−0.126 ***	−0.0546
	(0.0447)	(0.0432)	(0.0448)	(0.0435)
ln (*invention*)			−0.0248	−0.0675 ***
			(0.0206)	(0.0207)
No. of observations	1848	1850	1843	1845
R-squared	0.815	0.875	0.812	0.875
City fixed effect	YES	YES	YES	YES
Year fixed effect	YES	YES	YES	YES

Note: The robust standard errors are reported in parentheses. All regressions control for the city fixed effects and the year fixed effects. Superscripts ***, **, * denotes significances at the 1%, 5%, 10% levels, respectively.

**Table 6:** Robustness checks—sample adjustment.

Variable	(1)	(2)	(3)	(4)
	ln (*SO_2_*)	ln (*Wastewater*)	ln (*SO_2_*)	ln (*Wastewater*)
*policy*	−0.160 ***	−0.102 ***	−0.226 ***	−0.135 ***
	(0.0581)	(0.0375)	(0.0559)	(0.0379)
ln (*grp*)	0.109	0.723 ***	−0.145	0.239 **
	(0.169)	(0.164)	(0.139)	(0.101)
ln (*patent*)	−0.0473	−0.103 **	−0.0805 **	−0.0392
	(0.0358)	(0.0423)	(0.0315)	(0.0285)
ln (*open*)	−0.00276	−0.0266	−0.0385 **	−0.0213
	(0.0184)	(0.0197)	(0.0189)	(0.0166)
ln (*pop*)	−0.347	−0.442	−0.156	0.0565
	(0.264)	(0.295)	(0.208)	(0.173)
*indus*	0.461	1.544 ***	1.539 ***	0.296
	(0.444)	(0.465)	(0.381)	(0.279)
*greenrate*	−0.108 **	−0.0939	−0.0904 **	−0.0397
	(0.0440)	(0.0605)	(0.0449)	(0.0424)
No. of observations	1311	1313	1699	1701
R-squared	0.864	0.883	0.807	0.876
City fixed effect	YES	YES	YES	YES
Year fixed effect	YES	YES	YES	YES

Note: The robust standard errors are reported in parentheses. All regressions control for the city fixed effects and the year fixed effects. Superscripts ***, ** denotes significances at the 1%, 5% levels, respectively.

**Table 7:** Mechanism analysis—industry structure.

Variable	(1)	(2)	(3)	(4)
	*Si*	*Si*	*Ti*	*Ti*
*policy*	−0.0335 ***	−0.0310 ***	0.0177 ***	0.0174 ***
	(0.0055)	(0.0052)	(0.0045)	(0.0043)
Control variables	NO	YES	NO	YES
No. of observations	1921	1862	1921	1862
R-squared	0.830	0.876	0.865	0.883
City fixed effect	YES	YES	YES	YES
Year fixed effect	YES	YES	YES	YES

Note: The robust standard errors are reported in parentheses. All regressions control for the city fixed effects and the year fixed effects. Superscripts *** denotes significances at the 1% level.

**Table 8:** Mechanism analysis—fiscal capacity.

Variable	(1)	(2)	(3)
	ln (*Exp*)	ln (*Rev*)	*Gaprate*
*policy*	0.0950 **	0.108 ***	−0.0671
	(0.0394)	(0.0396)	(0.0758)
Control variables	YES	YES	YES
No. of observations	1863	1863	1863
R-squared	0.925	0.924	0.516
City fixed effect	YES	YES	YES
Year fixed effect	YES	YES	YES

Note: The robust standard errors are reported in parentheses. All regressions control for the city fixed effects and the year fixed effects. Superscripts ***, ** denotes significances at the 1%, 5% levels, respectively.

## Data Availability

The data were mainly collected from the *China City Statistical Yearbooks* and the database of the CNRDS platform (https://www.cnrds.com/).

## Appendix A

**Table A1:** Effect of the policy on other pollution emissions.

Variable	(1)	(2)	(3)
	ln (*NH*)	ln (*COD*)	ln (*PM_2.5_)*
*policy*	−0.248 **	−0.194 **	−0.0036
	(0.118)	(0.0945)	(0.0145)
ln (*grp*)	0.613 **	0.443 **	−0.0240
	(0.293)	(0.213)	(0.0317)
ln (*patent*)	−0.232 ***	−0.0198	−0.0421 ***
	(0.0888)	(0.0603)	(0.0077)
ln (*open*)	−0.0316	−0.0849 **	0.00154
	(0.0485)	(0.0334)	(0.0046)
ln (*pop*)	1.898 ***	0.916 **	−0.0428
	(0.538)	(0.446)	(0.0519)
*indus*	−1.269	−0.147	0.0266
	(0.842)	(0.572)	(0.085)
*greenrate*	0.244	0.0613	0.0079
	(0.592)	(0.485)	(0.0188)
No. of observations	1661	1663	1862
R-squared	0.786	0.851	0.954
City fixed effect	YES	YES	YES
Year fixed effect	YES	YES	YES

Note: *NH* represents industrial ammonia nitrogen emission, COD represents industrial chemical oxygen demand emission, and PM_2.5_ represents fine particles with a diameter of 2.5 μm or less. The robust standard errors are reported in parentheses. All regressions control for the city fixed effects and the year fixed effects. Superscripts ***, ** denotes significances at the 1%, 5% levels, respectively.
